# Prevalence and associated factors of frailty among community dweller older adults living in Gondar town, northwest, Ethiopia: a community based cross-sectional study

**DOI:** 10.1186/s12889-023-16201-w

**Published:** 2023-07-07

**Authors:** Mihret Dejen Takele, Kedir Sany, Kefale Getie, Dechasa Imiru Wayessa, Gashaw Jember, Melese Gobezie, Yohannes Abich, Alemu Kassaw Kibret

**Affiliations:** Department of Physiotherapy, School of Medicine, College of Medicine and Health Sciences, university of Gondar, Gondar, Ethiopia

**Keywords:** Prevalence, Frailty, Older adults, Ethiopia

## Abstract

**Background:**

Frailty is a multidimensional geriatric condition that increases vulnerability to stressors, increases the risk of negative health outcomes, and lowers quality of life in older people. However, little attention has been paid to frailty in developing countries, particularly in Ethiopia. Therefore, the aim of the study was to investigate the prevalence of frailty syndrome and the sociodemographic, lifestyle, and clinical factors associated with it.

**Methods:**

A community-based cross-sectional study design was conducted from April to June 2022. A total of 607 study participants were included using a single cluster sampling technique. The Tilburg frailty indicator, which is a self-reported schedule for assessment of frailty, required respondents to answer ‘yes’ or ‘no’ and the total attainable score ranged from 0 to 15. An individual with a score of ≥ 5 considered frail. Data were collected by interviewing the participants using a structured questionnaire, and the data collection tools were pre-tested before the actual data collection period to check for the accuracy of responses, language clarity, and appropriateness of the tools. Statistical analyses were performed using the binary logistic regression model.

**Results:**

More than half of the study participants were male, and the median age of the study participants was 70, with an age range of 60–95 years. The prevalence of frailty was 39% (CI 95%, 35.51–43.1). In the final multivariate analysis model, the following factors associated with frailty were obtained: older age (AOR = 6.26 CI (3.41–11.48), presence of two or more comorbidities (AOR = 6.05 CI (3.51–10.43), activity of daily life dependency (AOR = 4.12 CI (2.49–6.80), and depression (AOR = 2.68 CI (1.55–4.63) were found to be significant factors.

**Conclusion and recommendations:**

Our study provides epidemiological characteristics and the risk factors of frailty in the study area. Efforts to promote physical, psychological, and social health in older adults are a core objective of health policy, especially for older adults aged 80 and above years, and those with two or more comorbidities.

**Supplementary Information:**

The online version contains supplementary material available at 10.1186/s12889-023-16201-w.

## Background

According to the World Health Organization report, the global population of older adults aged 60 years or more is expected to rise to around 2 billion by 2050 [1]. This aging population in Ethiopia in 2015 was 5.2 million, accounting for more than 5% of the total population, and is expected to rise to 6.1% in 2030 and 10.4% in 2050 [2].

Human aging is a dynamic and progressive natural process which is depends on interacting hereditary, biological, social, environmental, historical and cultural factors that determine the quality of life of an older individual [3]. The concept of frailty is defined by the inability to maintain homeostasis in response to even minor stressors, in which these changes accumulate to the degree where they may cause increased levels of vulnerability and a decline in quality of life among the older adult population [4]. Fried et al. defined frailty as five key areas indicating compromised energetics, i.e., low grip strength, low energy, slow walking speed, low physical activity, and/or unintentional weight loss, for the diagnosis of frailty [4]. Very briefly, frailty means infirmity, weakness, and a lack of physical and mental strength [5].

Frailty is a multidimensional geriatric condition that increases vulnerability to stressors, increases the risk of negative health outcomes, and lowers quality of life, making it one of the most difficult challenges for health care in an aging society [6–8].

Prevalence of frailty is present in millions of older adults worldwide however, the global prevalence of frailty is not yet known, partly because frailty research has predominantly been done in high-income countries [9]. In a systematic review conducted in 2012, the weighted prevalence of frailty in high income countries was 10.7% [10]. Another systematic and meta-analysis of studies from different populations in low and middle-income countries based on 2007 World Bank income category has reported that the prevalence of frailty among older adults varied from 4% in China to 51% in Cuba [11]. A systematic review and meta-analysis of population-level studies across 62 countries revealed that the prevalence of frailty ranged from 75% among those aged ≥ 65 years in Romania and 91% among centenarians in Italy to < 1% in Denmark for individuals aged ≥ 50 years [12].

Frail older adults are at increased risk of premature death and various negative health outcomes, including falls, fractures, disability, and dementia, all of which could result in poor quality of life and increased cost and use of health care resources, such as emergency department visits, hospitalization, and institutionalization [13–16]. Studies were done among community-dwelling older adults have showed that the healthcare costs of frail individuals are sometimes several-fold higher than those of non-frail counterparts [17, 18].

Risk factors for the onset of frailty or frailty progression span a wide range of aspects and conditions, covering sociodemographic, clinical, lifestyle-related, and biological domains [19]. Previous studies conducted among older adults showed that increasing age, female gender, living alone, low educational level, low income status, depression, morbidity, and low level of physical activity are very much associated factors with frailty [20–25].

In fact, frailty is regarded as a pre-disability state and, therefore, if early detection of frailty and identifying risk factors on time could guide public health and preventive strategies, in particular when these risk factors are potentially modifiable by specific interventions [9].

Generally frailty is considered as a dynamic condition, with proper interventions’ frailty can be altered or prevent much adverse health outcome in the older people leading to good mental and physical health and satisfaction quality of life [26, 27], however, without proper intervention, deterioration for older adults may occur and become exposed too much adverse health outcome and poor quality of life among older adults [28].

Most of the research has been conducted in developed countries and a difference in results exists among studies and recommends widespread research in this context, especially in developing countries. However, studies to determine the prevalence of frailty in developing countries like Ethiopia is not stated. Therefore, relevant research is required to clearly state the magnitude and associated factors of frailty among older adults in Ethiopia. Addressing the burden and factors contributing to frailty is important to early detection, prevention and treatment strategy on older populations. Therefore, this study aims to determine the prevalence and associated factors of frailty among community-dwelling older adults living in Gondar town, Northwest Ethiopia.

## Methods

### Study design and setting

A community based cross-sectional study was conducted from April to June 2022. The study was conducted in Gondar town, Amhara regional state, Northwest Ethiopia. The city is located in central Gondar zone, Amhara regional state, 748 km Northwest of Addis Ababa, Ethiopia capital, and about 180 km from Bahir Dar, Amhara regional state’s capital. Gondar is among one of the ancient and largely populated cities in the country. It has an altitude of 12˚360 N 37˚280E and a longitude of 12.60˚ N 37.467˚E with an elevation of 2133 m above sea level. Gondar town has 25 kebeles (the smallest administrative units in Ethiopia). According to the Gondar statistics agency’s 2021/22 projection from 2007 population census data, the total population of Gondar town was estimated at 390,000 more than half of the population were women and, 6879 were older adults [29]. The town has one comprehensive specialized hospital and eight health centers; they are providing health services to the population.

### Population and sample size

The source population was all population of community dwelling older adult age 60 years and above living in Gondar town. Older adults, aged 60 years and above in selected kebeles (which is the smallest administrative unite in Ethiopia) during the study period, has been the study population. Older adults aged 60 years and above who were permanent residents (≥ 6 month) in the selected kebeles were included.

### Sample size determination

The sample size was determined using single population proportions formula assuming, 50% anticipated prevalence of frailty, since there was no study conducted before in Ethiopia, a 95% confidence interval, and a 5% marginal error.

n = sample size, Z_α/2_ (1.96) = critical value at 95% confidence interval, p = expected estimates of prevalence value of frailty (50%), d = Margin of sampling error (5%).

n = (Z_α/2_)² p (1-p)/d², n= (1.96)^2^ × (0.5) (0.5)/ (0.05)² = 384.16 = 385.

By considering a design effect of 1.5 and 10% non-response rate, the minimum adequate final sample size was 636. But because of the effect of cluster sampling, a total of 670 older adults were interviewed from a total of 645 household.

### Sampling technique and procedure

**Fig. 1:**
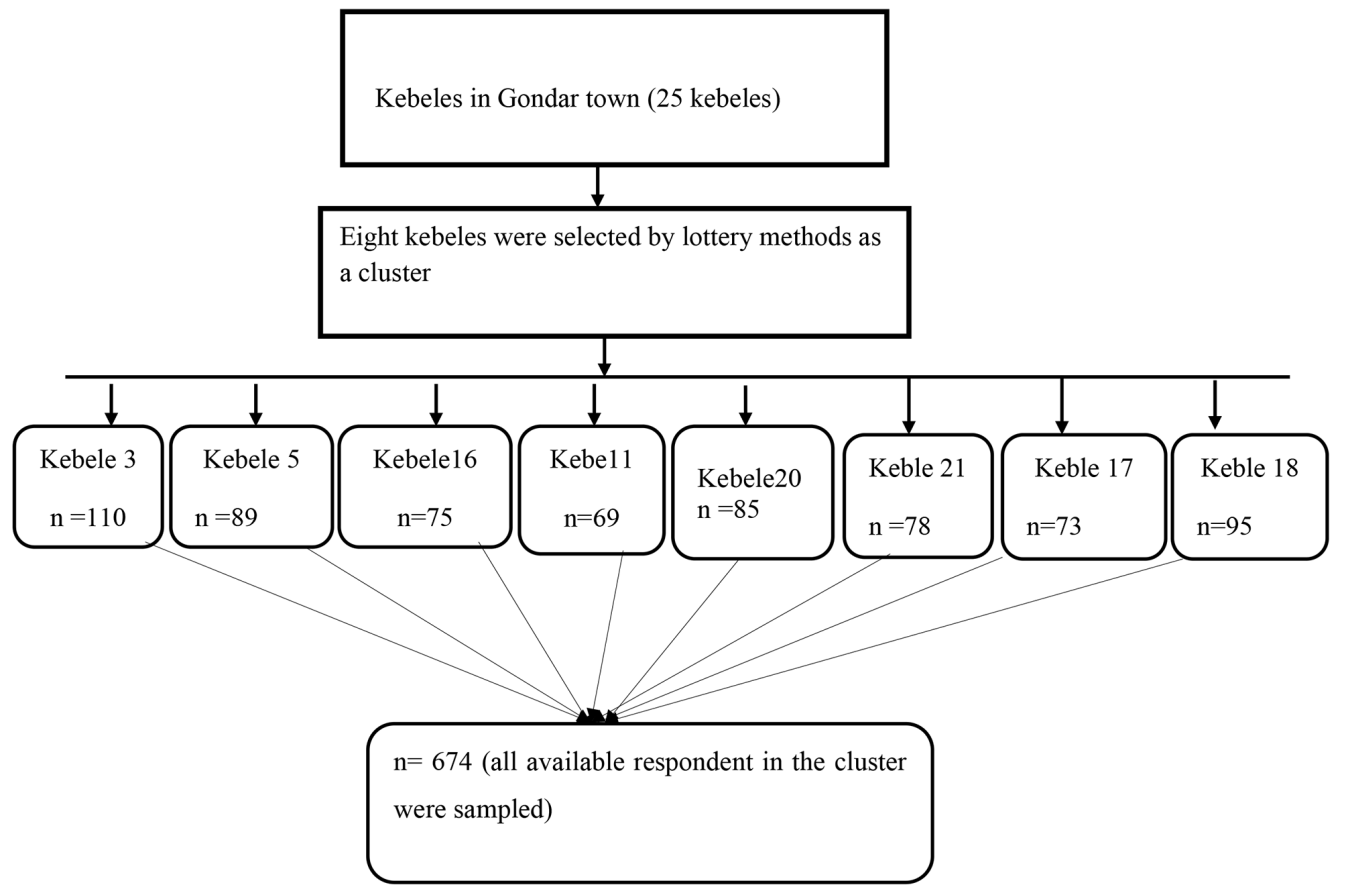
Flow chart diagram showing sampling technique and procedure

Gondar town has 25 kebeles. Eight kebeles were selected by lottery method. A single stage cluster sampling technique was used to select study participants. All eligible older adults in the selected cluster were interviewed in their household (Fig. 1)

### Variables

#### Dependent variables

The dependent variable is the self-reported assessment of frailty. Respondents were required to answer ‘yes’ or ‘no’ and the total attainable score ranged from 0 to 15. An individual with a score of ≥ 5 considered frail.

#### Independent variables

Sociodemographic related variables’ such as sex, age, marital status, educational level, income status, and living arrangement; clinical related variables’ hospitalization, multi-morbidity, ADL dependency, and depression; and behavioral-related variables’ **s**moking, alcohol consumption, and physical activity.

### Methods of data collection

After obtained permission from ethical review committee from university of Gondar collage of medicine and health science, house to house visit was done. Face to face interview was taken from study participants using a predesigned pretested structured schedule with the following domains.

1. Sociodemographic characteristics;
2. Lifestyle related characteristics.
3. Clinical related characteristics.
4. Geriatric depression (Geriatric depression scale, short form (GDS).
5. Frailty (Tilburg frailty indicator (TFI).
6. Activities of daily life (ADL questionnaire).

The other detailed contents of the questionnaire were developed from previous literature, and the questionnaire was modified based on all the variables that directly meet the objective of the study. It was prepared in an English version and translated to the Amharic language back to English to ensure consistency by language experts. Data collection was done by four trained health extension workers and two physiotherapist supervisors.

### Operational definition

1. **Tilburg frailty indicator part B**: It is a self-reported schedule for assessment of frailty through its three important components, such as physical, psychological, and social. Eight questions regarding physical component, four questions on psychological component, and three questions on social component were asked. Respondents were required to answer ‘yes’ or ‘no’ and the total attainable score is ranged from 0 to 15. An individual with a score of ≥ 5 considered to be frail [30].
2. **Katz index of independence**: is used to assess the functional status of older adults. The index ranks adequacy of performance in the six functions of (bathing, dressing, toileting, transferring, continence, and feeding). Its interpretation of scored is given yes = 1/no = 0 for independence in each of the six functions of items, and a score of 6/6 indicates full function, a score of 4/6 indicates moderate impairment and if its score of 2/6 or less indicates severe functional impairment and the attainable score will be 0 to 6. An individual with score of ≤ 5 was taken as ADL dependence [31].
3. **Geriatric Depression Scale short form (GDS)**: Was used to screen for depressive symptoms in this study. The 15 items in the GDS-SF were extracted from the original 30-item GDS. Respondents were required to answer ‘yes’ or ‘no’ to the 15 statements that describe either a positive or a negative emotion/condition. Attainable score ranges from 0 to 15 and an individual with a score of ≥ 5 considered to be depressed [32].

### Statically analysis

The collected data was entered into Epidata and exported, coded and analysis was done using Statistical Package for the Social Sciences (SPSS) Version 26. An analysis of binary logistic regression was used to identify the factors that would predict the outcome variable. For both bivariate and multivariable logistic regression analyses, a cutoff p-value of 0.25 and 0.05 was considered a significant level, respectively. Prior to determining the final independent predictor variables for frailty, the bivariate logistic regression analysis was done, and variables that were determined to be statistically significant were included in the multiple logistic regression analysis. Variables with a p value of < 0.05 at 95% confidence interval (CI) and their odds ratio (OR) were used to interpret the findings of the final model.

## Results

A total of 607 older adults were included in this study, making a response rate of 90%. Among the total respondents more than half of the study participants 312 (51.4%), were male and the median age of the study participants were 70, an inter-Quartile Range (IQR) 65–80, age range from 60 to 95 years (See in Table 1).

**Table 1:** Sociodemographic characteristics of community dweller older adults living in Gondar town, Northwest, Ethiopia, 2022 (n = 607)

Variables	Frequency (n)	Percentage (%)
**Sex**		
Male	312	51.4
Female	295	48.6
**Age(in years)**		
60–69	261	43.0
70–79	180	29.7
≥ 80	166	27.3
**Educational status**		
Illiterate or Primary	91	15.0
Secondary	307	50.6
Tertiary or higher	207	34.4
**Marital status**		
Unmarried /divorce/widow	199	32.8
Married	408	67.2
**Living arrangement**		
Living with children/other family Living with spouse only	215 247	35.4 40.7
Living alone	145	23.9
**Income status**		
≤ 1500	291	47.9
1501–3500	112	18.5
≥ 3501	204	33.6

### Clinical related characteristics

More than one third, 219 (36.1%) of study participants had two or more comorbidities and 161 (26.5%) of study participants, had a history of hospitalization in the past one year. Almost one third of study participants 208, (34.1%) were ADL dependent and 406 (66.9%) were depressed (See in Table 2).

**Table 2:** Clinical related characteristics of the study participant of community dweller older adults living in Gondar town, Northwest Ethiopia, 2022 (n = 607)

Variables	Frequency (n)	Percentage (%)
**Morbidity**		
None	247	40.7
One	141	23.2
Two	219	36.1
**Hospitalizations**		
Yes	161	26.5
No	446	73.5
**ADL dependency**		
Yes	208	34.5
No		65.7
**Depression**		
Yes	406	66.9
No	201	33.1

### Lifestyle related characteristics

From total participants, 223 (36.7%) of the study participants’ were physical inactive. In regard to smoking status, 51 (8.4%) of the participants were a smoker, and 142 (23.4%) were alcoholic (See in Table 3).

**Table 3:** lifestyle related characteristics of the study participant among community dweller older adults living in Gondar town, Northwest Ethiopia, 2022 (n = 607)

Variables	Frequency(n)	Percentage (%)
**Physical activity level (in minutes per week)**		
< 150	223	36.7
≥ 150	384	63.3
**Current smoker**		
Yes	51	8.4
No	556	91.6
**Alcoholic**		
Yes	142	23.4
No	465	76.6

### Prevalence of frailty

The overall prevalence of frailty in this study was found to be 39% (CI 95%, 35.5–43.1). Among those who had developed frailty, the majority of study participants 136 (46.1%) were female and regarding age category, 125(75.3%) of them were aged 80 and older. Likewise, the majority of the study participants 51(56.0%) were Illiterate or Primary educational status, and nearly two thirds of the study participants 128 (64.3%) were unmarried/divorced/widowed are (shown in Table 4).

**Table 4:** Sociodemographic characteristic and frailty among community dweller older adults living in Gondar town, Northwest Ethiopia, 2022 (n = 607)

Variables	Frailty	
	Yes n (%)	No n (%)
**Sex**		
Male	101(32.4)	211(67.6)
Female	136(46.1)	159(53.9)
**Age category in year**		
60–69	49(18.8)	212(81.2)
70–79	63(35.0)	117(65)
≥ 80	125(75.3)	41(24.7)
**Educational status**		
Illiterate or Primary	51(56.0)	40(44.0)
Secondary education	133(43.3)	174(56.7)
Tertiary or higher	53(25.4)	156(74.6)
**Marital status**		
Married	109(26.7)	299(73.3)
Unmarried/divorce/widowed	128(64.3)	71(35.7)
**Living arrangement**		
Living with children/other family	105(48.5)	110(51.2)
With spouse only	48(19.4)	199(80.6)
Living alone	84(57.9)	61(42.1)
**Income status**		
≥ 3501	37(18.1)	167(81.9)
1501–3500	35(31.3)	77(68.7)
< 1500	165(56.7)	126(43.3)

### The associated factors of frailty among older adults

In bivariate logistic regression analysis (unadjusted) variables such as sex, age, marital status, income status, morbidity, hospitalization, ADL dependency and depression were significantly associated with frailty. In multivariate logistic regression (adjusted) variables such as, age 80 and older (AOR = 6.26 CI (3.41–11.48), having two or more morbidity (AOR = 6.05 CI (3.51–10.43), ADL dependency (AOR = 4.12 CI (2.49–6.80) and depression (AOR = 2.68 CI (1.55–4.63) were significantly associated with frailty are (Shown in Table 5).

**Table 5:** Bivariate and multivariable logistic regression analysis of associated factors among community dweller older adults living in Gondar town, Northwest Ethiopia, 2022 (n = 607)

	Frailty			OR 95%CI		
**Variables**	Yes	No		COR (95% CI)		AOR 95%CI
**Sex**						
Male	101	211		**1**		**1**
Female	136	159		1.78(1.28–2.48)		1.03(0.64–1.66)
**Age in years**						
60–69	49	212	**1**		**1**	
70–79	63	117	2.32 (1.5–3.60)		1.47(0.83–2.59)	
≥ 80 and older	125	41	13.19(8.24–21.1)		6.26 (3.41–11.48) *	
**Marital status**						
Unmarried/divorce/widowed	128	71	4.94(3.43–7.11)		1.47(0.83–2.59)	
Married	109	299	**1**		**1**	
**Income status**						
≤ 1500	165	126	5.91(3.86–9.04)		1.64(0.90–2.97)	
1501–3500	35	77	2.05(1.20–3.50)		1.45(0.72–2.91)	
≥ 3500	37	167	**1**		**1**	
**Morbidity**						
None	64	183	**1**		**1**	
One	42	99	1.21(0.76–1.92)		1.43(0.77–2.66)	
Two	131	88	4.25(2.87–6.30)		6.05 (3.51–10.43) *	
**Hospitalization**						
Yes	76	85	1.58(2.40–4.80)		1.05(0.61–1.80)	
No	161	285	**1**		**1**	
**ADL dependency**						
Yes	153	55	10.43(7.05–15.42)		4.12(2.49–6.80) *	
No	84	285	**1**		**1**	
**Depression**						
Yes	208	201	5.38(3.52–8.23)		2.68(1.55–4.63) *	
No	32	169	**1**		**1**	

Note 1 = Reference category, CI = confidence interval * statistically significant at P < 0.05

## Discussion

The aim of this study was to assess the prevalence and associated factors of frailty among community dweller older adult living in Gondar town. The overall prevalence of frailty among older adults living in Gondar town in this study was 39% (CI 95%, 35.5–43.1). This finding indicates that frailty is a high public health burden and health problem among community dweller older adults living in Gondar town. The results of the present study revealed that frailty among older adults is significantly associated with age 80 and older, having two or more morbidity, being ADL dependent and depression.

The prevalence of frailty in our study (39%) was in line with a study conducted in West India (38.8%) [5] and Netherlands (40.2%) [33]. This might be due to similar study methodology and measuring tool (TFI) used.

However, it was lower compared with studies conducted in Italy among centenarians (91%) [34], in Romania (75%) [35], Cuba (51%) [36] and south India (63%) [23]. This discrepancy might be due to a different frailty measurement tool and the study participant’s age. For example, the study participants’ ages ranged from 99 to 113 years in Italy and Romania, aged 65 to 95 years. This is confirmed by the fact that with an ageing population, there is a growing interest in frailty [37]. In addition, unlike our study, where study participants were recruited from the public, study participants in Cuba were recruited from a geriatric medical facility. This is supported by additional research showing that residents of medical care facilities had a higher prevalence of frailty than people living in the general population [10, 38]. Similarly, the fact that the study population in south India was made up primarily of rural dweller perhaps be the cause of the disparity between our study and the study conducted there. Older people in rural areas are said to have lower incomes, lower levels of education, and less access to health care and insurance, all of which contribute to poorer health [39].

Our study reported higher prevalence of frailty compared with study done in the USA (9.1%) [40], Saudi Arabia (21.4%) [41] and China (9.9%) [39]. This difference might be due to the method of identifying frailty tool, socioeconomic status, and health service variation of the study participants. In addition, unlike our study Fried’s frailty criteria [4], which assess primarily the physical aspect of the research participants, were used to measure frailty in the studies conducted in the United States, Saudi Arabia and China. However, in the current investigation, frailty was evaluated using (Tilburg’s frailty indicator) which is a multidimensional method that took the study participants’ physical, psychological, and social dimensions into account. Another argument could be elderly individuals living in high-income countries have different socioeconomic statuses, are more conscious of healthy living, are financially secure, and have access to superior healthcare [42].

According to this study, participants 80 years of age and older were 6.26 times more likely to experience frailty than those between the ages of 60 and 69. This study’s findings were consistent with research from South India, Colombia, Saudi Arabia, and Indonesia that found significant association between frailty and age 80 and beyond [23, 41, 43, 44]. The interactions between particular systems that raise the risk of frailty, like inflammation and endocrine dysregulation, and physiologic changes associated with advancing age may be the cause of this [45]. In addition, physiologic changes in old age may lead to sarcopenia and a higher risk of frailty [46].

The results of this study also showed that people who had two or more comorbidities were 6.05 times more likely to develop frailty than those who did not. Similar to this, our study’s finding, were supported by studies from Brazil, Spain, Singapore, and the United States, that revealed a substantial relationship between frailty and comorbidity [40, 47–49]. The accumulating effects of medical conditions and other deficiencies in old age may be the cause of unfavorable health outcomes like reduced quality of life, disability, prolonged hospital admissions, complex pharmaceutical regimens, and susceptibility to frailty [50, 51].

This study showed that persons with ADL dependence were 4.12 times more likely to acquire frailty than participants without ADL dependence. Our study’s findings, which were also corroborated by research from the USA, Brazil, and West India, showed that the presence of ADL dependence in older persons was strongly associated with frailty [5, 40, 52]. This could be because older persons with ADL dependence engage in less physical exercise, which raises their risk of frailty [46].

Furthermore, this study found that persons with depression were 2.68 times more likely to become feeble than participants without depression to experience frailty. According to this study’s findings, depression in older adults was the highest risk factor for frailty, which is consistent with research from European, China and Iran [25, 39, 53]. Given that depressed people frequently lose weight, become inactive, and subsequently lose muscle mass, strength, and tolerance to exercise, factors leading to an increase in cytokines which is closely linked to the onset of frailty, and could be the hypothesis that depressive symptoms trigger frailty from a biological point of view [46].

### Limitations and the strengths of the study

The findings of our study are unlikely to be transferable to other contexts because we only enrolled older persons who resided in metropolitan communities. Because of the cross-sectional design, the cause of frailty cannot be proven. Despite these drawbacks, it is a groundbreaking study that fills a significant evidence vacuum about frailty in Ethiopia, particular in the study area.

## Conclusion

Our study provides epidemiological characteristics and the risk factors of frailty in the study area; the findings indicate guiding actions that minimize adverse effects in the ageing process. Efforts to promote physical, psychological, and social health in older adults are a core objective of health policy, especially for older adults aged 80 and above and those with two or more comorbidity.

## Electronic supplementary material

Below is the link to the electronic supplementary material.

Supplementary Material 1

## Data Availability

Since this is a funded work, the raw data is the property of the University of Gondar. The datasets used and/or analyzed during the current study are available from the corresponding author on reasonable request at **mihretdejen2017@gmail.com**.

## Abbreviations

ADL: Activity of Daily Living
AGS: American Geriatrics Society
AOD: Adjusted Odd Ratio
BGS: British Geriatrics Society
CDC: Communicable Disease Control
CGA: Compressive Geriatrics Society
I: Confidence Interval
COR: Crude Odd Ratio
FI: Frailty Index
GFI: Groningen Frailty Index (GFI)
QOF: Quality of Life
SPSS: Statistical Package for Social Software
TFI: Tilburg Frailty Indicators
USA: United States of America
WHO: World Health Organization
