# Supplementary material for: Prevalence and associated factors of frailty among community dweller older adults living in Gondar town, northwest, Ethiopia: a community based cross-sectional study
Source: BMC Public Health. 2023 Jul 7;23:1309. doi: 10.1186/s12889-023-16201-w (PMC10329322; doi:10.1186/s12889-023-16201-w)
Supplement: Supplementary file 1 — Supplementary Material 1 [file 12889_2023_16201_MOESM1_ESM.docx]

Fill or circle the space that provide your answer

| **Part one: Scio demographics information** | | | |
| --- | --- | --- | --- |
| No | Question | Possible answer | skip |
| 101 | What is the sex of the respondent? | 1. Male 2. Female |  |
| 102 | Ho w old are you? | ________________ in year |  |
| 103 | What is your marital status? | 1. Unmarried 2. Marred 3. Divorce 4.Widow |  |
| 104 | What is your education level? | 1. None 2. primary education 3. Secondary education. 4. Higher professional or university education |  |
| 105 | How is your living arrangement | 1. Living alone 2.Living with children/ others family 3.With spouse only |  |
| 106 | What is your current monthly income in Ethiopian birr (ETB)? | ---------birr cent------ |  |

|  | **Part two: Clinical related factors** |  | |  |
| --- | --- | --- | --- | --- |
| 201 | Do you have chronic disease? | 1.Yes | 2.No | HF1 |
| 202 | If you say yes question HF1 how many chronic disease do you have? | ___________________________ | |  |
| 203 | Do you have a history hospitalization in past one year? | 1.Yes | 2.No |  |

|  | **Part three: Measuring activity of daily life by using Katz Index of Independence** | | |  |
| --- | --- | --- | --- | --- |
|  | Activities |  | |  |
| 301 | Do you ask supervision, direction or personal assistance when you bathing? | 1.Yes | 2.No |  |
| 302 | Do you ask supervision, direction or personal assistance when you dressing? | 1.Yes | 2.No |  |
| 303 | Do you ask supervision, direction or personal assistance when your toileting? | 1.Yes | 2.No |  |
| 304 | Do you asking supervision, direction or personal assistance when you move and out of bed or moving from bed to chair? | 1.Yes | 2.No |  |
| 305 | Can you control when your urine or bowels are coming out until you reach the toilet? | 1.Yes | 2.No |  |
| 306 | Do you move food from plate into mouth without help? | 1.Yes | 2.No |  |

|  | **Part four: Measurement of depression using GDS-SF** |  |  |  |
| --- | --- | --- | --- | --- |
| 401 | Are you basically satisfied with your life? | 1.Yes | 2.No |  |
| 402 | Have you dropped many of your activities and interests? | 1.Yes | 2.No |  |
| 403 | Do you feel that your life is empty? | 1.Yes | 2.No |  |
| 404 | Do you often get bored? | 1.Yes | 2.No |  |
| 405 | Are you in good spirits most of the time? | 1.Yes | 2.No |  |
| 406 | Are you afraid that something bad is going to happen to you? | 1.Yes | 2.No |  |
| 407 | Do you feel happy most of the time? | 1.Yes | 2.No |  |
| 408 | Do you often feel helpless? | 1.Yes | 2.No |  |
| 409 | Do you prefer to stay at home, rather than going out and doing new things? | 1.Yes | 2.No |  |
| 410 | Do you feel you have more problems with memory than most? | 1.Yes | 2.No |  |
| 411 | Do you think it is wonderful to be alive now? | 1.Yes | 2.No |  |
| 512 | Do you feel pretty worthless the way you are now? | 1.Yes | 2.No |  |
| 413 | Do you feel full of energy? | 1.Yes | 2.No |  |
| 414 | Do you feel that your situation is hopeless? | 1.Yes | 2.No |  |
| 415 | Do you think that most people are better off than you are? | 1.Yes | 2.No |  |

|  | **Part five : Information on lifestyle factors** | | | |
| --- | --- | --- | --- | --- |
| 501 | Have you engaged in any type of regular physical exercise (Aerobic exercise, muscle strengthening exercise and balance exercise). | 1.Yes | 2.No | BF01 |
| 502 | If you say yes question BF01 how often do you do exercise per week | ……………….. minute/day  ………………..day/week | | |
| 503 | Do you smoke cigarette | 1.Yes | 2.No | BF03 |
| 504 | If you answer yes for BF03, how many cigarettes do you smoke per day? | ________Cigarette per day |  |  |
| 505 | Do you drink alcohol? | 1.Yes | 2.No | BF05 |
| 506 | If you answer yes for BF05, How much alcohol drink per day | 1. ≥ 2 bottle beer/day 2. < 2 bottle beer/day | | |

|  | **Part six: Measuring frailty using Tilburg Frailty Indicator (TFI)** | | | |  |
| --- | --- | --- | --- | --- | --- |
|  | **Physical components** |  | |  |  |
| 601 | Do you feel physically healthy? | 1. No | | 0.Yes |  |
| 602 | Have you lost a lot of weight recently without wishing to do so? (‘a lot’ is: 6 kg or more during the last six months, or  3 kg or more during the last month) | 0. No | | 1.Yes |  |
| 603 | Do you experience problems in your daily life due to difficulty in walking? | 0. No | | 1.Yes |  |
| 604 | Do you experience problems in your daily life due to difficulty maintaining your balance? | 0. No | | 1.Yes |  |
| 605 | Do you experience problems in your daily life due to poor hearing? | 0.No | | 1.Yes |  |
| 606 | Do you experience problems in your daily life due to poor vision? | 0. No | | 1.Yes |  |
| 607 | Do you experience problems in your daily life due to lack of strength in your hands? | 0. No | | 1.Yes |  |
| 608 | Do you experience problems in your daily life due to physical tiredness? | 0.No | | 1.Yes |  |
|  | **Psychological compensate** |  | |  |  |
| 609 | Do you have problems with your memory? | 0.No | Sometimes=0 | 1.Yes |  |
| 610 | Have you felt down during the last month? | 0.No | Sometimes=1 | 1.Yes |  |
| 611 | Have you felt nervous or anxious during the last month? | 0.No | Sometimes =1 | 1.Yes |  |
| 612 | Are you able to cope with problems well? | 1.No |  | 0.Yes |  |
|  | **Social component** |  | |  |  |
| 613 | Do you live alone? | 0.No |  | 1.Yes |  |
| 614 | Do you sometimes miss having people around you? | 0.No | 1.Sometimes | 1.Yes |  |
| 615 | Do you receive enough support from other people? | 1.No |  | 0.Yes |  |
